# Lung fibrosis in autoimmune diseases and hypersensitivity: how to separate these from idiopathic pulmonary fibrosis

**DOI:** 10.1007/s00296-021-05002-2

**Published:** 2021-10-04

**Authors:** Helmut Popper, Elvira Stacher-Priehse, Luka Brcic, Andreas Nerlich

**Affiliations:** 1 Medical University Graz, Diagnostic and Research Institute of Pathology, Neue Stiftingtalstr. 6, 8036 Graz, Austria; 2 Institute of Pathology, Asklepios-Fachkliniken Gauting, Gauting, Germany; 3 Department of Pathology, Teaching Hospital Munich-Bogenhausen, Munich, Germany

**Keywords:** Fibrosing pneumonia, Chronic autoimmune disease, Rheumatoid arthritis, Systemic sclerosis, Sjøgren syndrome, Systemic lupus, Chronic hypersensitivity pneumonia, UIP, IPF

## Abstract

Lung involvement in autoimmune diseases (AID) is uncommon, but may precede other organ manifestations. A diagnostic problem is chronicity presenting with lung fibrosis. A new category of interstitial pneumonia with autoimmune features for patients with clinical symptoms of AID and presenting with usual interstitial pneumonia (UIP) enables antifibrotic treatment for these patients. Hypersensitivity pneumonia (HP) and other forms of lung fibrosis were not included into this category. As these diseases based on adverse immune reactions often present with unspecific clinical symptoms, a specified pathological diagnosis will assist the clinical evaluation. We aimed to establish etiology-relevant differences of patterns associated with AID or HP combined with lung fibrosis. We retrospectively evaluated 51 cases of AID, and 29 cases of HP with lung fibrosis, and compared these to 24 cases of idiopathic pulmonary fibrosis (UIP/IPF). Subacute AID and HP most often presented with organizing pneumonia (OP), whereas chronicity was associated with UIP. Unspecified fibrosis was seen in a few cases, whereas NSIP pattern was rare. In 9 cases, the underlying etiology could not be defined. Statistically significant features differentiating chronic AID or HP from UIP/IPF are lymphocytic infiltrations into myofibroblastic/fibroblastic foci. Other features significantly associated with AID and HP were granulomas, isolated Langhans giant cells, and protein deposits, but seen in only a minority of cases. A combination of UIP with one of these features enabled a specific etiology-based diagnosis. Besides the antifibrotic drug regimen, additional therapies might be considered.

**Supplementary Information:**

The online version contains supplementary material available at 10.1007/s00296-021-05002-2.

## Introduction

Autoimmune diseases (AID) are heterogenous, some of them affecting joints, others involving blood vessels, but all can involve the lung [1–3]. They all have in common deregulation of the immune system resulting in auto-aggression against normal tissues [4–8]. In some diseases, a clinical diagnosis is usually straightforward as patients present with a typical clinical picture, e.g., skin affection and glomerular disease in systemic lupus erythematosus (systemic lupus), or joint inflammation in rheumatoid arthritis. Most of these AIDs can affect the lung. In a minority of cases, lung involvement can precede the classical symptoms delaying or impeding the diagnosis [9]. This can result in undetected and untreated pulmonary disease, which might progress into fibrosis. Diagnosis of hypersensitivity pneumonia (HP) is straightforward in the acute stage by an exposure anamnesis, typical undulating symptoms associated with allergen exposure, and morphologically by loose peripherally concentrated epithelioid cell granulomas combined with lymphocytic interstitial pneumonia (LIP), dominated by CD8^+^ T cells. Due to the increased sensitivity of CT scans and awareness of fibrosing pneumonia, more biopsies are performed, resulting in an increase of AID and HP with lung fibrosis.

Three classic patterns are recorded in AID and HP with pulmonary fibrosis: usual interstitial pneumonia (UIP), non-specific interstitial pneumonia (NSIP), and organizing pneumonia (OP). Unspecified fibrosis of the lung is rarely seen [1, 10–15]. UIP is also the pattern of idiopathic pulmonary fibrosis (IPF). A sequence of morphologic events is seen in UIP, starting with apoptosis of pneumocytes, followed by a repair by myofibroblasts, and regeneration of the epithelial layer, during which some epithelial cells differentiate into senescent cells [16–19]. Inflammatory cells will be absent or only be seen in cystic remodeled areas due to bacterial colonization. Senescent cells release inflammatory cytokines, stimulate the proliferation of myofibroblasts, and prolong the repair process, resulting in fibrosis. Different molecular abnormalities have been found in IPF as well as in UIP-associated AIDs, e.g., premature aging of pneumocytes due to mutations of telomere maintenance genes [20–22], mutations of surfactant apoprotein genes [23–25], contributing to prolonged inflammation, and promoter mutation in MUC5B [26, 27], which might reduce mucociliary clearance and disrupt regeneration [28].

As AID and HP associated with UIP clinically often cannot be attributed to a specific AID, the term interstitial pneumonia with autoimmune features (IPAF) has been created [29]. This term encompasses active and inactive AID with anti-nuclear antibodies but excludes HP. Morphologically different types of fibrosing pneumonia are included [30]. If UIP in IPF can be differentiated from UIP in AID and fibrosing HP has rarely been evaluated but might be of prognostic relevance [1, 12, 14, 31–34]]. Some publications showed that lymphoid hyperplasia is associated with systemic sclerosis, dermatomyositis/polyserositis, and rheumatoid arthritis. They also reported a different anatomical distribution for each of these AIDs and mentioned rheumatoid nodules as a distinctive feature for rheumatoid arthritis [34, 35].

Reports on survival of AID or HP with UIP pattern are divergent [14, 31, 33, 36–38]. Similar to IPF, both diseases progress stepwise. Here, we aimed to analyze the different patterns of AID and fibrosing HP in a retrospective series and compare this to IPF to identify features, which might allow an etiology-based diagnosis. We believe a more detailed pathological report over just a UIP pattern report will help in a better stratification of patients and concise discussion within the multidisciplinary team [39]. Additional therapies to the established antifibrotic treatment protocol might be considered.

## Materials and methods

From the lung tissue archive of the Institute of Pathology, Medical University of Graz, 113 cases were selected when any kind of lung fibrosis was present, and the initial clinical diagnosis and a clinical follow-up were available. We did not include end-stage diseases, such as seen in explant lung tissue, because a specific diagnosis often is not possible in these tissues.

There were 51 cases of clinically active AID, 29 cases of HP, and 24 clinically confirmed IPF cases based on the presence of UIP pattern. In seven cases, the final diagnosis was either AID or HP. In two cases, a final diagnosis of either IPF or AID could not be reached. In all these cases, a clinical diagnosis and clinical response to the pathological report was received either submitted with the tissue or after the pathological report was issued. Most cases were submitted for consultation. Fibrosis was seen in the form of UIP, fibrosing NSIP, OP, unspecific fibrosis, and airway-centered interstitial fibrosis (ACIF) (Suppl. Table 1).

### Definition of patterns is given in Suppl. Table 2

In UIP, we use the term myofibroblastic foci instead of fibroblastic focus because these cells are essentially myofibroblasts expressing myogenic markers; also, honeycombing is replaced by cystic remodeling, which can be seen in primary lobules, whereas radiologists by honeycombing describe cystic changes in secondary lung lobules. In ACIF, fibrosis extends from small airways to the periphery, combined with muscular hyperplasia and metaplasia of bronchial epithelia [40]. In contrast to NSIP, LIP is characterized by diffuse monomorphic infiltration of lymphocytes and plasma cells, with few scattered immunoblasts, with or without follicular hyperplasia of BALT (bronchus-associated lymphoid tissue), whereas in cellular NSIP histiocytes and macrophages combine with lymphocytes. Scattered and focal infiltrations of lymphocytes were not regarded as LIP but were described. Other features of AID and HP are histiocytic or epithelioid cell granulomas, amyloid or immune complex deposition with/without complement activation, isolated BALT hyperplasia, lymphocytic bronchitis/bronchiolitis, follicular bronchiolitis, peribronchial fibrosis (constrictive bronchiolitis), and hemorrhage—often mixed in different proportions. Amyloid was defined by a positive Congo red stain with green birefringence and by immunohistochemistry for amyloid A, or P. Immune complex deposition was verified by a positive immunohistochemical reaction for IGG and concomitant activation of complement 1q, 3c, and 5–9 complex in formalin-fixed and paraffin-embedded tissues.

The tissues were obtained by video-assisted thoracoscopy (open lung biopsy) or bronchoscopy-derived cryobiopsies. Hematoxylin and Eosin (H&E)-stained slides were available in all cases. In cases where a differential diagnosis of AID or HP was raised, immunohistochemistry for lymphocyte subtypes was performed, using antibodies for CD3, CD4, CD8, CD20, rarely for FOXP3, characterizing regulatory T cells. All cases were re-evaluated by HP and ES. The presence or absence of the following patterns was recorded for each case: myofibroblastic foci, cystic remodeling of lung lobules, spatial and temporal heterogeneity, fibrosis, lymphocytes infiltrating the myofibroblastic foci, hyperplasia of BALT, histiocytic and/or epithelioid granulomas, isolated Langhans giant cells, amyloid or immune complex deposition, complement activation, vasculitis, hemorrhage, vasculopathy (myxoid changes of the intima with few scattered lymphocytes), vascular sclerosis, neutrophils, eosinophils. Additional features not generally associated with fibrosing pneumonias were also recorded (e.g., chronic bronchitis/bronchiolitis and pleuritis, peribronchial fibrosis).

For statistical analysis, cases were separated into AID, HP, and IPF. The program Kaleidagraph (Synergy Software, v4.5) was used. A significance was stated if the *p* value was ≤ 0.05.

## Results

### Study cohort

There were altogether 51 cases with active AID, 29 cases of HP with fibrosis, and 24 confirmed UIP/IPF cases. 9 cases could not be assigned into these categories (Suppl. Tables 1 and 2).

### Histological findings in autoimmune disease

In 21 cases, a UIP pattern was seen, in two of them combined with OP; in 9 cases, UIP was combined with LIP pattern. In 10 cases, OP was prevalent, in 2 of them combined with LIP. In 9 cases, non-specific interstitial fibrosis was seen, in the majority combined with LIP. One case presented with fibrosing NSIP, and in one case, only BALT hyperplasia was found (Suppl. Table 3).

In 28 additional cases of AID, a more specific diagnosis could be established: rheumatoid arthritis in 10, systemic sclerosis in 10, systemic lupus in 2, Sjøgren’s disease in 2 cases, and one case each for Behcet disease, dermatomyositis, Goodpasture syndrome, and one case probably associated with autoimmune liver disease.

### Findings in hypersensitivity pneumonia

In 19 of 29 cases of HP, a UIP pattern was seen, combined with LIP in 12 cases. Five cases presented with OP, in four combined with LIP; 4 cases showed unspecified fibrosis, in 3 combined with LIP, ACIF was present in one case.

In 7 cases, a differential diagnosis of either AID or HP was made. (Suppl. Table 3). There were lymphocytic infiltrates within the myofibroblastic foci, however, no other additional features; for subtyping, the number of lymphocytes was too low.

### Findings in IPF

All 24 cases of IPF showed the typical UIP pattern without pronounced inflammation (Table 1, Suppl.Table3). In 2 additional cases, a differential diagnosis of either AID or IPF was rendered—these were cryobiopsies with few myofibroblastic foci and scattered lymphocytes outside the myofibroblastic foci.

**Table 1:** Features, which are helpful for the differentiation of autoimmune disease and fibrosing hypersensitivity pneumonia from idiopathic pulmonary fibrosis (presence of feature/pattern = 1, absence = 0); * denotes which of the entities are statistically compared

Features	AID	HP	IPF	Significance
Lymphocytes in myofibroblastic foci	Yes*(1) 0.91 ± 0.28	Yes (1)	No* (0) 0.16 ± 0.38	*p < 0.0001, AID vs IPF
Lymphocytes in myofibroblastic foci	Yes* (1)	Yes* (1)		*p = 0.92, AID vs HP
Granulomas or giant cells	Yes* (1)	Yes (1)	No* (0)	*p = 0.028, AID vs IPF
Granulomas or giant cells	Yes* (1) 0.66 ± 0.47	Yes* (1) 0.28 ± 0.45		*p < 0.001, HP vs AID
Hyperplasia of BALT	Yes* 0.48 ± 0.50	Yes** 0.32 ± 0.47	No* 0.0	*AID vs IPF *p* = 0.001; **HP vs IPF *p* = 0.023 AID vs HP *p* = 0.09
Amyloid or immune complex deposition	Yes*	No*	No	*AID vs HP *p* = 0.004
Predominance of CD8 lymphocytes	No	Yes	No	
Mixed lymphocytic infiltrations (CD4, CD8, CD20)	Yes	No	No	
Chronic pleuritis ± fibrin exudate if present	Yes	No	No	

### Different patterns in AID, HP, and IPF (see Table 1 and Suppl. Table 3)

**Fig. 1:**
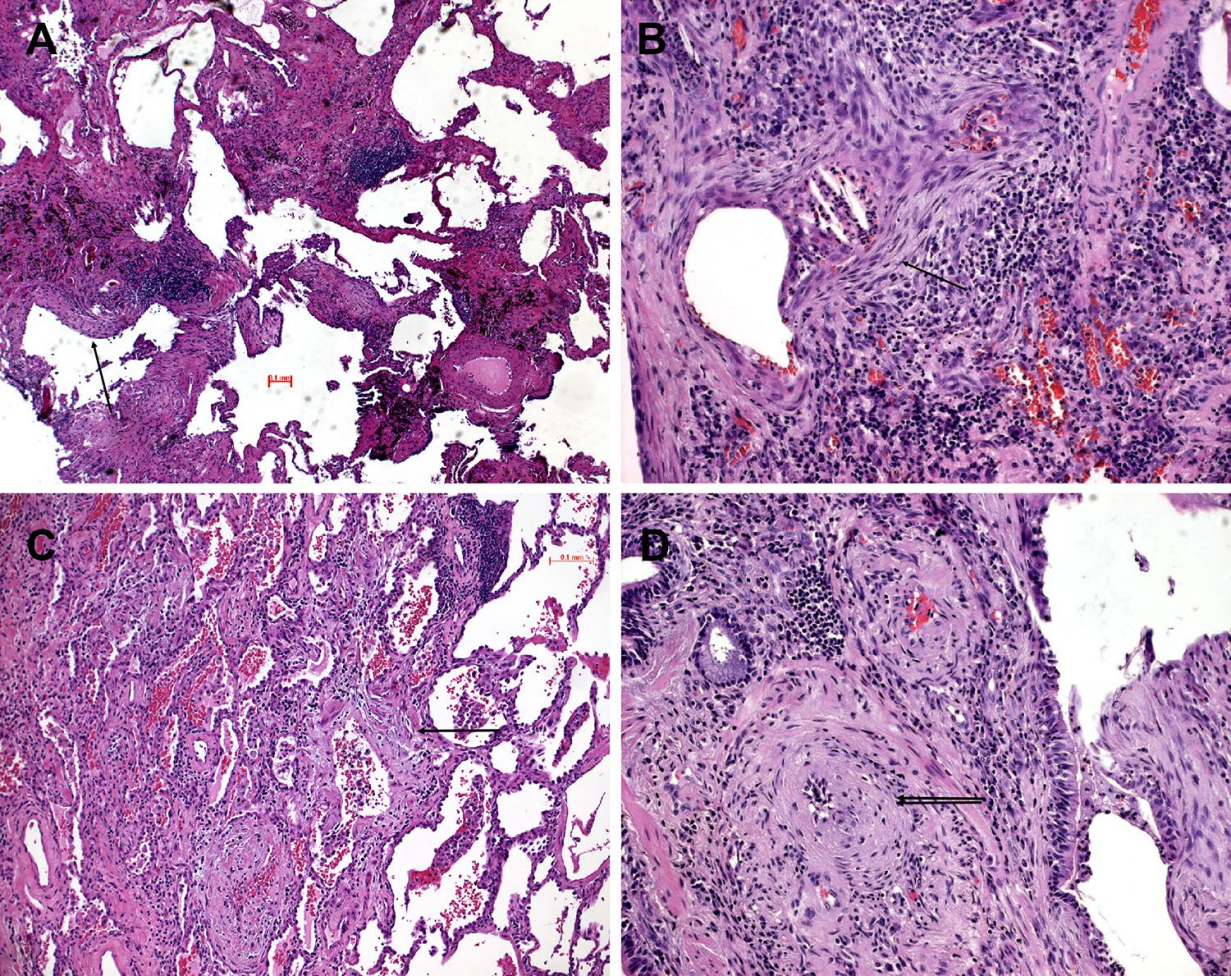
Systemic sclerosis illustrated; a UIP pattern was present with myofibroblastic foci (single arrows) and remodeling (**A**, **B**, **C**, **D**); in (**B**) the intimate association of lymphocytes with a myofibroblastic focus (single arrow) is a significant feature pointing to an immune disorder with UIP pattern. In (**D**), the myxoid changes of the intima (double arrow) are seen in a middle-sized pulmonary artery. H&E, bars 100, 50, 20 µm

**Fig. 2:**
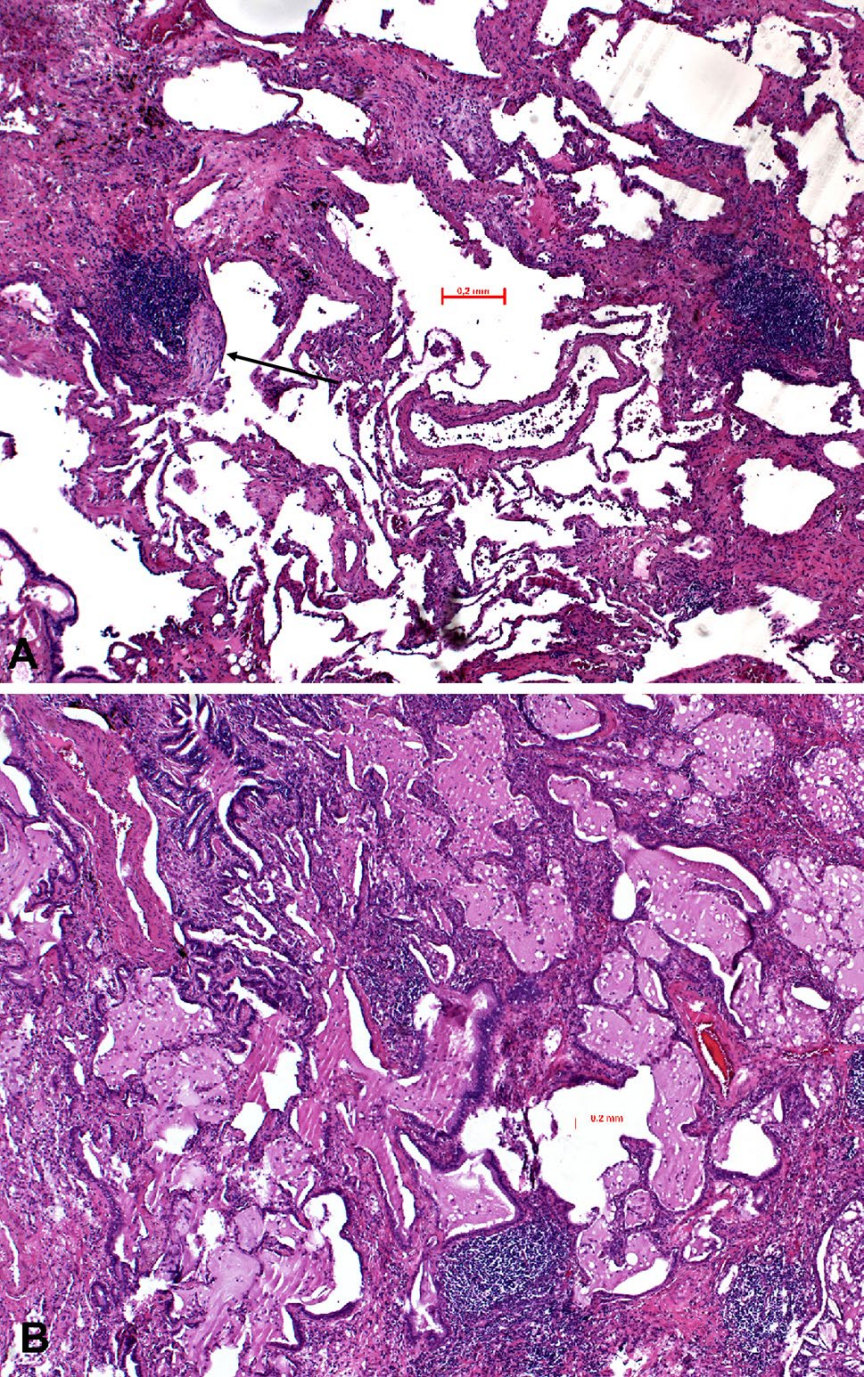
Hyperplasia of the bronchus-associated lymphoid tissue in two cases of systemic sclerosis with UIP pattern. In (**A**) myofibroblastic foci in addition to lymph follicles associated with bronchovascular bundles are seen (arrow); in (**B**) cystic remodeling of lung lobules with mucostasis are seen. H&E, bars 200 µm

**Fig. 3:**
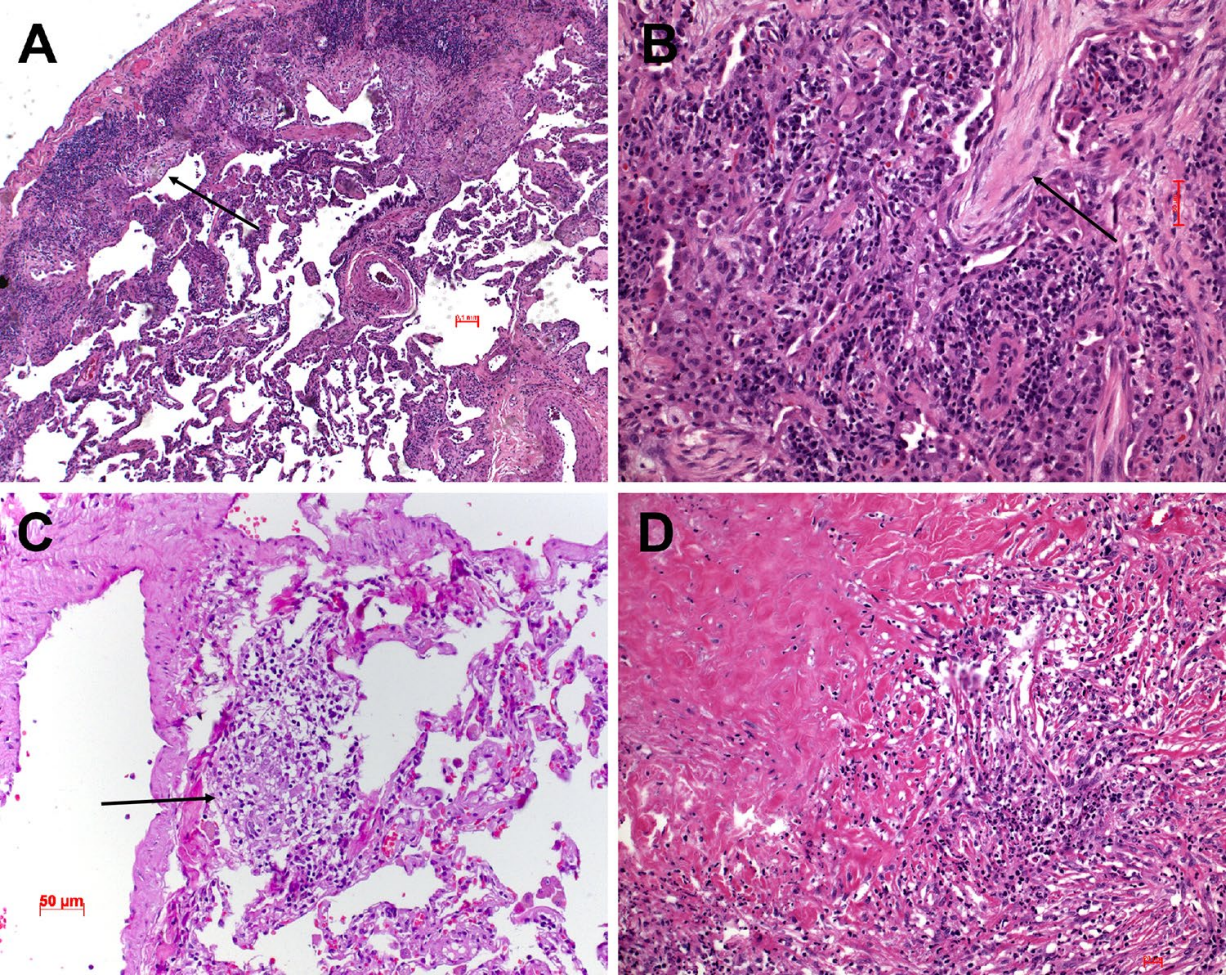
Rheumatoid arthritis with lung involvement, one case is illustrated; In **A,** a dense lymphoid infiltration is seen, myofibroblastic foci (arrows) are in (**A**) and (**B**) a loose granuloma (arrow) is shown in (**C**), and amyloid in (**D**).H&E, bars 100, 50 µm

**Fig. 4:**
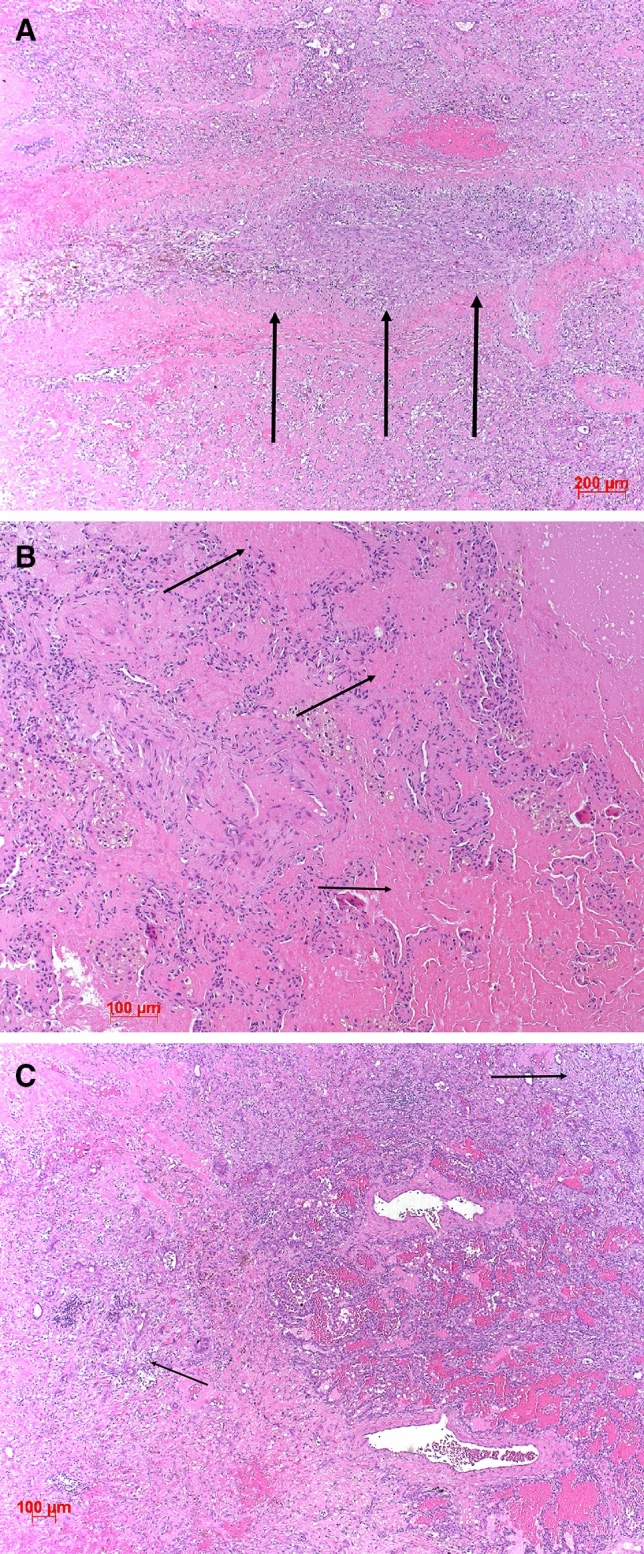
SLE illustrated; **A** shows arterial thrombosis in an organization (arrows), eosinophilic deposits (arrows) mimicking amyloid and unspecific fibrosis is seen in (**B**), and fibrosis focally OP (arrows) with hemorrhage in **C**). H&E, bars 200, 100 µm

The presence of lymphocytes infiltrating myofibroblastic foci in AID and HP (Fig. 1) was statistically significantly different from IPF (*p* < 0.0001, Table 1), whereas no significant difference was seen between AID and HP (*p* = 0.92). The presence of histiocytic/epithelioid cell granulomas and/or Langhans giant cells in AID versus IPF was significantly different, as granulomas were absent in all IPF cases (*p* = 0.028, Table 1); however, granulomas were present in a minority of AIDs. Granulomas or isolated Langhans cells were more often encountered in HP compared to AID, their presence favoring HP (*p* < 0.001). The presence of BALT hyperplasia (Fig. 2) favored AID as well as HP (*p* = 0.001 and *p* = 0.023, respectively) when compared to IPF; no significant difference was seen between AID and HP (*p* = 0.09; Table 1). There was no deposition of amyloid or autoimmune complexes (Figs. 3, 4) in IPF and HP, which rendered this feature significant for AID (*p* = 0.004, Table 1). But again, only a small number of AID cases presented with deposits. Other features such as chronic bronchitis/bronchiolitis, vasculitis, hemorrhage, and degenerative changes of the intima (vasculopathy) were seen in few cases; therefore, a statistical analysis was not performed. In contrast to other reports, we rarely encountered peribronchial or peribronchiolar fibrosis in AID and HP (9 and 3 cases each).

### Differences of AID versus HP by Immunohistochemistry

In cases of HP and AID, where a sufficient number of lymphocytes could be evaluated, a predominance of CD8^+^ lymphocytes were seen in HP, whereas in AID CD20^+^ B cells, CD4^+^, and CD8^+^ T cells were present in different proportions; scattered FOXP3^+^ regulatory T cells were seen in HP—these were scarce in AID. Inflammatory infiltrates within the pleura always favored AID (Table 1).

**Fig. 5:**
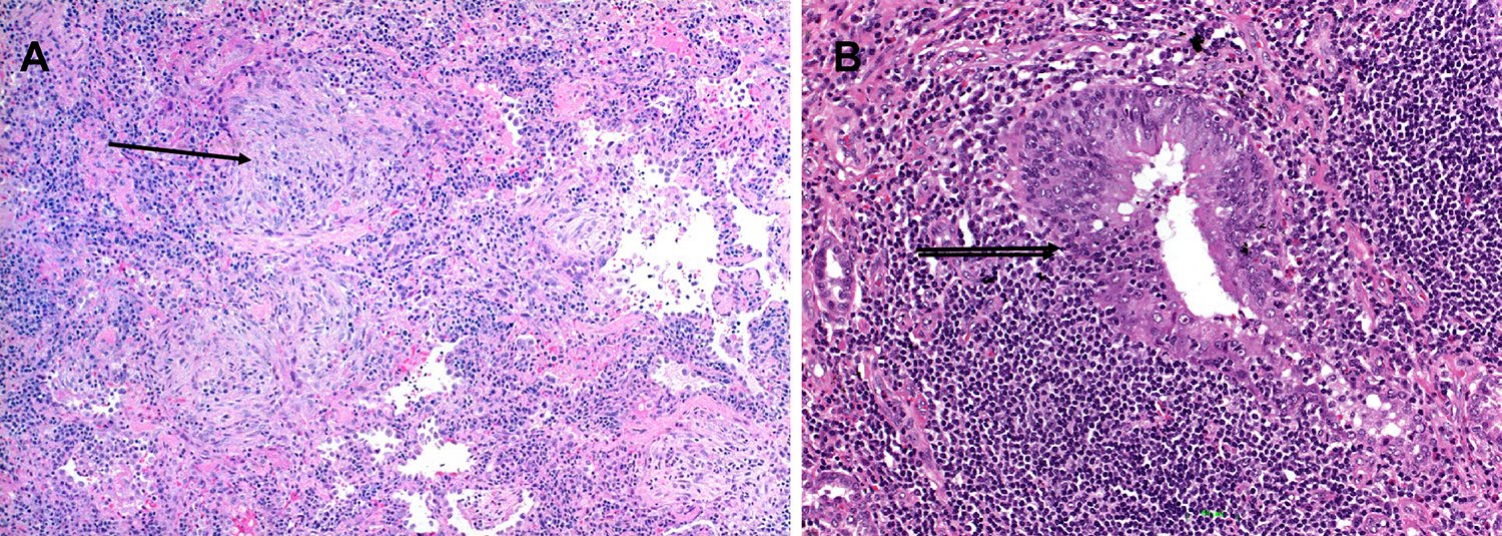
Sjøgren’s disease illustrated; in (**A**), organizing pneumonia (arrow) and dense lymphocytic infiltrations are seen, focally (**B**) showing a lymphoepithelial lesion (double arrow). H&E, bars 100 and 20 µm

In several additional AID cases, a more specific diagnosis could be suggested to the clinicians (Tables 1, 2, and 3). Examples were cases of rheumatoid arthritis presenting with combinations of UIP, LIP, granulomas, and amyloid deposits (Fig. 3). A combination of arterial thrombosis, immune complex deposits, complement activation, and hemorrhage pointed to systemic lupus (Fig. 4). A LIP pattern combined with OP and lymphoepithelial lesions with predominant CD8^+^ T cells favored Sjøgren’s disease (Fig. 5). Finally, UIP combined with LIP, hyperplasia of BALT, and myxoid changes of the intima of pulmonary arteries were suggestive of systemic sclerosis (Fig. 1). Goodpasture disease could be suggested due to alveolar hemorrhage, fibrosis, and the proof of linear immunoglobulin deposits at the basal membrane of alveolar septa and capillaries combined with complement activation. In Behcet disease, a rheumatoid disorder was suggested because of large fibrotic areas, few ill-formed epithelioid cell granulomas, amyloid deposits, and scattered lymphocytic infiltration (Suppl. Figure 1).

**Table 2:** Features seen in autoimmune diseases. The more morphologic features are combined, the better the diagnosis can be specified

Patterns present	Rheumatoid arthritis	SLE	SSc	Dermato-myositis	Sjøgren	Fibrosing HP
UIP pattern	Yes	No	Yes	Yes	No	Yes
NSIP pattern	Yes	No	Yes	Yes	No	Yes
OP pattern	Yes	Yes	Yes	Yes	Yes	Yes
Unspecific Fibrosis	Yes	Yes	No	Yes	Yes	No
Lymphocytic infiltrations or LIP	Yes	Yes	Yes	Yes	Yes	Yes
Giant cells	Yes	No	No	Yes	No	Yes
Lymphoid hyperplasia	Yes	No	Yes	Yes	Yes	Yes
Epithelioid or histiocytic cell granulomas	Yes	No	No	Yes	No	Yes
Alveolitis						
Amyloid deposition	Yes	No	Yes	Yes	Yes	No
Immune complex deposition	Yes	Yes	Yes	Yes	No	No
Complement activation	Yes	Yes	No	No	?	No
Vasculitis						
Vasculopathy	No	Yes	Yes	No	No	No
Vascular sclerosis	No	No	Yes	No	No	No
Alveolar hemorrhage, fresh and old	No	Yes	No	No	Yes	No
Neutrophils	Yes	Yes	Yes	Yes	No	No
Eosinophils	Yes/no	No	No	Yes	No	Yes
Bronchiolitis and/or specific forms thereof	Yes	No	Yes	Yes	Yes	Yes
LE phenomenon	No	Yes	No	No	No	No

The more morphologic features are combined, the better the diagnosis can be specified

**Table 3:** Combinations of features which might allow a more specific diagnosis for certain AIDs

Rheumatoid arthritis	UIP, LIP, granulomas, amyloid, CD4/CD8/CD20 present
Systemic Lupus	arterial thrombosis, hemorrhage, pleuritis, immune complex deposition
Systemic sclerosis	UIP, LIP, hyperplasia of BALT, vasculopathy
Chronic/subacute Sjøgren	LIP, OP, lymphoepithelial lesions, CD8^+^ dominance
Behcet disease and other rheumatoid diseases	Fibrosis, granulomas, amyloid deposition, lymphocytic infiltrations, ill-formed granulomas

## Discussion

Fibrosing pneumonias are histopathologically differentiated into UIP, NSIP, and OP. International guidelines suggest not to perform biopsies when clinical and radiological features favor UIP/IPF [41]. However, new studies showed that the radiologic features of UIP are not specific enough to make a diagnosis of IPF. Wright et al. evaluated 23 cases and found that features of peribronchiolar metaplasia and giant cells or granulomas were in favor of HP and excluded IPF—features which can only be assessed in biopsies [42]. A multidisciplinary team discussion could not solve even one-third of their cases. Similarly, Churg pointed to other features favoring HP over IPF, such as upper-lobe predominance, giant cells or granulomas, and peribronchiolar metaplasia [43]. A few reports have focused on AID/connective tissue diseases, showing a fibrosing pattern very similar to IPF [34, 44, 45]. In the studies of Ito and Kim, several cases had to be reclassified from IPF into connective tissue disease. Even genetic alterations of surfactant and telomerase genes have been reported in AID similar to IPF, illustrating that these molecular alterations cannot assist in the differential diagnosis [46, 47]. Whereas the NSIP pattern almost exclusively is associated with AID or HP, the UIP pattern has a wider range of differentials. Positive anti-nuclear antibodies (ANA) are not always present in AID with lung fibrosis, and CT scans might discriminate IPF from AID only in cases with unusual distribution patterns [48]. If AID with UIP pattern has a better overall survival remains uncertain, and therefore studies separating these from IPF are warranted.

This study demonstrated that a pathological analysis of additional patterns to UIP, NSIP, or OP could provide more information about an underlying etiology. Lymphocytes infiltrating myofibroblastic foci are indicators of immune disease, either AID or HP. Other combinations of UIP with granulomas and/or giant cells, BALT hyperplasia, and protein deposits are also characteristic of immune disorders. Based on these findings, the pathological report can exclude IPF. The issued report can further strengthen the discussion in a multidisciplinary team.

Even more, due to specific patterns in some AIDs, the pathologic report can provide a preference for one of the AIDs, as shown here in roughly half of these cases (Table 1, 3). Rheumatoid arthritis with lung involvement can present with UIP or NSIP, often combined with LIP in the former. Pathognomonic histiocytic granulomas are seen especially in seropositive forms of RA. In rheumatoid arthritis, deposition of immune complexes, as well as amyloid, is common. Very large idiotypic–anti-idiotypic immune complexes with granulomatous reaction are most often encountered in rheumatoid arthritis less in systemic lupus [6, 49, 50]. Therefore, if a combination of these patterns is seen in a biopsy, rheumatoid arthritis can be suggested. In the authors’ experience, systemic lupus rarely presents with UIP, but often a combination of OP, unspecific fibrosis, thrombosis, and deposition of immune complexes is seen. In systemic lupus, these antigen–antibody complexes activate complement, which can be proven by immunohistochemistry. Systemic sclerosis is known for its high numbers of autoantibodies and circulating immune complexes. In our cases, a UIP pattern with hyperplasia of BALT and myxoid changes of the pulmonary arteries with scattered intimal lymphocytes was commonly seen and did allow the suggestion for systemic sclerosis. Sjøgren’s disease is characterized by an aggressive lymphocytic infiltration of the mucosa of bronchi and bronchioles, mimicking lymphoepithelial lesions. After excluding MALT lymphoma, a combination of OP and LIP with a CD8^+^ Tcell infiltration (in our cases) and lymphoepithelial lesions are suggestive for Sjøgren’s disease.

Fibrosing HP can, in some instances, be differentiated from AID. Protein deposits help to rule out HP, whereas scattered giant cells and ill-formed granulomas favor HP. In contrast to other reports, we could not see any significant difference between AID and HP with respect to BALT hyperplasia [51]. NSIP was relatively rare in our cases. NSIP and an isolated OP pattern were associated preferentially with AIDs, as previously reported [52], whereas ACIF was only seen in HP [40]. However, the OP pattern was combined with UIP in some cases, and more important, LIP was present in several cases.

For several years, cryobiopsies are preferred over open lung biopsies in diagnosing fibrosing pneumonias. These biopsies are useful in all cases where a clinical and radiological diagnosis already favors IPF, AID, or HP, respectively. However, in those cases with unusual clinical and radiological patterns, a video-thoracoscopic biopsy is superior. The distribution of patterns as described above can be very focal, involving different lung segments or even lobes. This is also seen on CT scans, where honeycombing might be seen in one focus and ground-glass opacities in another. A larger piece of tissue will increase the likelihood of sampling all these different patterns.

## Conclusion

We have shown that a pathological analysis can provide more than just IPAF diagnosis, which does not include fibrosing HP and encompasses acute disease. In our diagnostic workup, we should dig for the underlying etiology. In some cases, we might be able to discern AID from HP. This might provide additional clues for treatment. Even in those cases where the pathological report can only exclude IPF, this would enable clinicians to investigate additional treatment options. Although antifibrotic treatment is now recommended for any kind of UIP regardless of the underlying etiology, additional treatment options might be considered in cases of AID and HP.

In the authors’ experience, a biopsy should be performed not only in cases where CT scan findings are inconclusive [53]. Lynch recommended a re-review of IPF cases on a regular basis, as the diagnosis might change. In our consultation praxis, we have seen cases initially diagnosed as IPF by CT scan and clinical presentation, which later turned into AID. A careful pathological analysis can provide much more information and might help clinicians stratify their patients early on. There are several limitations of our study. One is the small sample size, and another also is due to the retrospective analysis performed here. Despite these limitations, our study underlines the necessity for lung biopsies in AID and HP. In future studies, tissues analysis will enable us to uncover molecular drivers and modifiers of autoimmune diseases and possibly find new targets for treatment.

## Supplementary Information

Below is the link to the electronic supplementary material.

Supplementary file1 (TIF 5581 KB) Behcet disease; extensive fibrosis is seen in (A), eosinophilic deposits in (B, C) mimicking amyloid; in (C), there is an area of necrosis with an ill-formed histiocytic granuloma; in (D) focal dense lymphocytic infiltrations are seen, myxoid changes of a large pulmonary artery and eosinophilic deposits. H&E, bars 400, 200, 50μm.

Supplementary file2 (DOCX 59 KB)

Supplementary file3 (DOCX 20 KB)

Supplementary file4 (DOCX 22 KB)

## Data Availability

All data have been included into the manuscript.
