# Supplementary figures and images for: Lung fibrosis in autoimmune diseases and hypersensitivity: how to separate these from idiopathic pulmonary fibrosis
Source: Rheumatol Int. 2021 Oct 4;42(8):1321–30. doi: 10.1007/s00296-021-05002-2 (PMC9287245; doi:10.1007/s00296-021-05002-2)

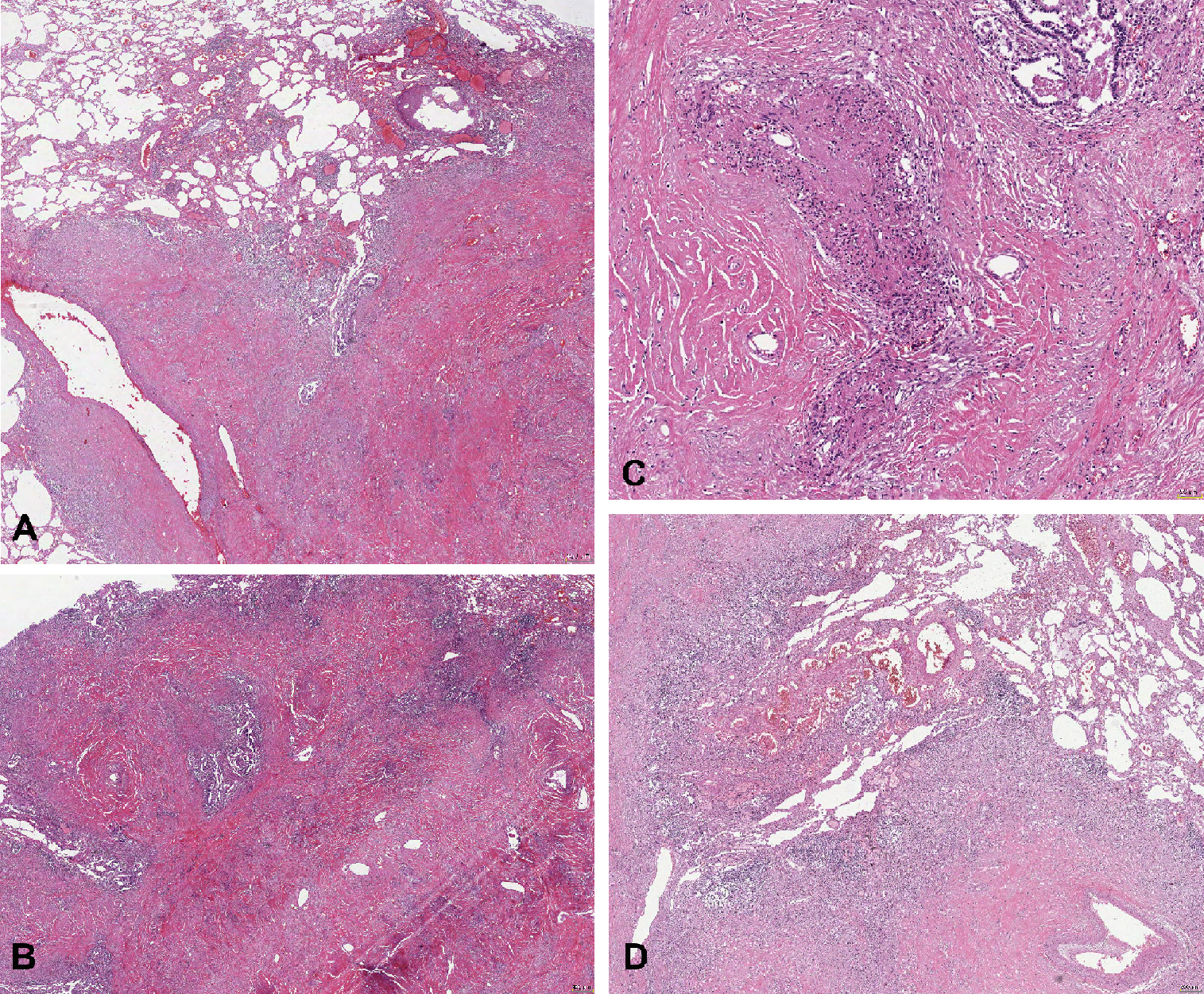

Supplement: Supplementary file 1 — Supplementary file1 (TIF 5581 KB) Behcet disease; extensive fibrosis is seen in (A), eosinophilic deposits in (B, C) mimicking amyloid; in (C), there is an area of necrosis with an ill-formed histiocytic granuloma; in (D) focal dense lymphocytic infiltrations are seen, myxoid changes of a large pulmonary artery and eosinophilic deposits. H&E, bars 400, 200, 50μm. [file 296_2021_5002_MOESM1_ESM.tif]
