# Supplementary material for: Lung fibrosis in autoimmune diseases and hypersensitivity: how to separate these from idiopathic pulmonary fibrosis
Source: Rheumatol Int. 2021 Oct 4;42(8):1321–30. doi: 10.1007/s00296-021-05002-2 (PMC9287245; doi:10.1007/s00296-021-05002-2)
Supplement: Supplementary file 2 — Supplementary file2 (DOCX 59 KB) [file 296_2021_5002_MOESM2_ESM.docx]

Supplementary Table 1: Clinical data, gender, age of the patients; abbreviations: CB=cryobiopsy, OLB/VATS= open lung biopsy; OP= organizing pneumonia, NSIP= non-specific interstitial pneumonia, UIP= usual interstitial pneumonia, AID= autoimmune disease, unspecified, ConstrBr= constrictive bronchitis/bronchiolitits, HP= hypersensitivity pneumonia,

| Case No | Age | Gender | Submitted clinical Diagnosis | Type of biopsy | Fibrosis pattern | Additional pattern | Final diagnosis |
| --- | --- | --- | --- | --- | --- | --- | --- |
| 12-24228 | 43 | m | Tumor, ILD | OLB | OP | LIP | AID and drug reaction |
| 06-60245 | 24 | M |  |  | NSIP fibrotic |  | AID |
| 08-116044 | 49 | M | Bilateral reticulo-nodular infiltrations, mediastinal lymphadenopathy, dyspnea on exertion, restrictive changes in spirometry | OLB | UIP, OP | OP | AID |
| 08-38083 | 60 | M | NSIP vs UIP | OLB | UIP | granulomas | AID |
| 08-63362 | 68 | M | ILD fibrosing, UIP? | OLB | UIP | RBILD | AID |
| 09-38314 | 69 | F? | COPD, tumor | OLB | OP | granulomas | AID |
| 10-115927 | 46 | M | UIP, improvement on corticosteroids | CB | UIP |  | AID |
| 10-94568 | 46 | M | Sarcoidosis, fibrosis | OLB | UIP | RBILD | AID |
| 11-103154 | 70 | F | Fibrosing alveolitis, hyper-eosinophilic syndrome? | OLB | UIP | pneumonia | AID |
| 11-46624 | 67 | F | Hypoxia, ILD, IPF? | OLB | UIP |  | AID |
| 13-59811 | 48 | M | ILD | CB | UIP | LIP | AID |
| 13-87984 | 55 | F | ILD emphysema | CB | UIP | LIP, chronic pleuritis | AID |
| 14-33764 | 57 | M | Suspicious for tumor | OLB | Fibrosis | LIP | AID |
| 14-33765 | 63 | M | COPD, bronchopneumonia, lung fibrosis? | OLB | UIP | LIP, chronic pleuritis | AID |
| 15-34490 | 73 | M | UIP/IPF? | CB | UIP | lymphocytic | AID |
| 18-27380 | 74 | M | ILD | CB | UIP | Lymphocytic chronic pleuritis | AID |
| 18-47334 | 64 | M | Adenocarcinoma, ILD, UIP? | OLB | UIP | Lymphocytic, chronic pleuritis, giant cells | AID |
| 18-51581 | 71 | M | Polymyalgia Rheumatica, Myelodysplastic syndrome/Steroid therapy | CB | UIP | Lymphocytic RBILD | AID |
| 18-60903 | 51 | M | No clinical data submitted | CB | UIP | Chronic pleuritis | AID |
| 14-48267 | 49 | M | ILD right LL | CB | UIP | LIP | AID |
| 14-38603 | 54 | M | Status post pneumonia; radiologically persistent infiltration left side | OLB | OP |  | AID subac? |
| 11-93534 | 54 | F | Recurrent chronic bronchitis, asthma | OLB | Fibrosis | ConBr, LIP | AID |
| 15-18858 | 60 | M | Lung fibrosis, pleuritis | OLB | UIP | lymphocytic | AID |
|  |  |  |  |  |  |  |  |
| 15-36617 | 72 | F | Lung fibrosis | OLB | UIP | LIP, chronic pleuritis | Rheumatoid arthritis |
| 17-101727 | 59 | M | Rheumatoid arthritis? | CB | UIP | LIP, follicular Br | Rheumatoid arthritis |
| 17-40236 | 47 | F | Breast carcinoma Olaparib therapy, lung fibrosis before therapy, high anti-SCL70, no signs of scleroderma | OLB | UIP | Lymphocytic chronic pleuritis CD4>8 | Rheumatoid arthritis |
| 17-44757 | 75 | F | NSIP radiologic; investigation for rheumatoid disease, suspicion for IgG4-disease, but not confirmed | OLB | UIP | LIP, chronic pleuritis follicular Br | Rheumatoid arthritis |
| 13-63573 | 54 | M | ILD? | OLB | UIP | RBILD, chronic pleuritis | Rheumatoid arthritis |
| 16-64572 | 40 | F | Miliary TBC, hilar lymphadenopathy | OLB | fibrosis | Granulomas hyalin deposits | Rheumatoid arthritis |
| 16-69402 | 77 | M | Churg-Strauss? | OLB | UIP | LIP ossification | Rheumatoid arthritis |
| 18-43892 | 76 | M | ILD | CB | fibrosis |  | Rheumatoid arthritis |
| 05-21897 | 64 | F | Sjogren syndrome, COPD | OLB | fibrosis | amyloid | Rheumatoid arthritis |
| 10-128899 | 79 | F | Unclear etiology, ANCA-, Sjogren, HP? | OLB | OP | Granulomas, LIP | Rheumatoid arthritis |
| 07-128466 | 22 | M | ILD, oxygen dependent | OLB | Constr.Br.CB |  | Sjogren |
| 14-29954 | 66 | F | Lymphoma? several nodules in lung | OLB | Fibrosis, OP | Cytotoxic lymphocytic | Sjogren |
| 10-18250 | 64 | F | LHCH? Siderosis | OLB | OP CB | granulomas | Systemic sclerosis |
| 17-82173 | 58 | M | ILD, UIP? AID? | OLB | UIP | Pleuritis chronic | Systemic sclerosis |
| 11-102694 | 58 | M | Radiologic NSIP, EAA ruled out by anamnesis | CB | UIP | Fibros Br ConB | Systemic sclerosis |
| 05-63129 | 24 | M | Systemic sclerosis? | CB | UIP | lymphocytic | Systemic sclerosis |
| 08-37926 | 55 | M | UIP and OP | OLB | UIP |  | Systemic sclerosis |
| 11-10084 | 43 | M | Fibrosis, viral infection? | OLB | UIP |  | Systemic sclerosis |
| 12-59248 | 67 | F | Arteritis temporalis | OLB | UIP | emphysema | Systemic sclerosis |
| 13-83663 | 58 | M | HIV, DAD? | OLB | UIP | LIP, RBILD | Systemic sclerosis |
| 14-29956 | 69 | M | CVD? | OLB | UIP |  | Systemic sclerosis |
| 11-51612 | 58 | M | ILD with reticular densities | OLB | UIP | DAD in org, ConBr | Systemic sclerosis |
| 12-54945 | 28 | M | Pneumonia, embolic disease, fever | OLB | OP | Chronic pleuritis thrombosis | Systemic Lupus |
| 12-42494 | 46 | F | SLE | OLB | OP | LIP granulomas | Systemic Lupus |
| 09-131989 | 45 | M | Dermatomyositis | OLB | OP UIP | OP | Dermatomyositis |
|  |  |  |  |  |  |  |  |
| 17-11578 | 43 | M | Mb Behcet/Mb Chron | OLB | OP fibrosis | granulomas | Behcet |
| 17-82512 | 29 | M | Mb Crohn, autoimmune hepatitis | OLB | Fibrosis | LIP giant cells | AID, Hepat? |
| 06-104509 | 43 | F | Hemosiderosis GP, asthma | OLB | Fibrosis, OP |  | Goodpasture |
|  |  |  |  |  |  |  |  |
| 15-31205 | 84 | M | Fibrosis and nodules | OLB | UIP | Lymphocytic giant cells | Fibrosing HP |
| 17-9343 | 55 | M | HP? | CB | UIP | LIP, chronic pleuritis RBILD | Fibrosing HP |
| 12-11898 | 64 | M | Sarcoidosis | OLB | fibrosis | granulomas | Fibrosing HP |
| 12-24672 | 58 | M | ILD, EAA? Left UL | OLB | UIP | granulomas | Fibrosing HP |
| 12-78030 | 63 | F | Foreign body aspiration? | OLB | UIP | granulomas | Fibrosing HP |
| 13-40336 | 51 | M | Nickel exposure | OLB | UIP | LIP | Fibrosing HP |
| 14-17832 | 74 | M | ILD with reticular densities | CB | UIP | LIP, granulomas | Fibrosing HP |
| 14-30982 | 59 | M | ILD | OLB | UIP | LIP | Fibrosing HP |
| 14-48596 | 59 | M | ILD | OLB | UIP | granulomas | Fibrosing HP |
| 16-18366 | 54 | M | IPF | OLB | UIP | LIP granulomas | Fibrosing HP |
| 16-22413 | 65 | M | Allergy or autoimmune disease? | OLB | UIP | LIP granulomas | Fibrosing HP |
| 16-47665 | 24 | F | Systemic sclerosis? | CB | UIP | LIP | Fibrosing HP |
| 17-1122 | 68 | M | ILD? | OLB | UIP | granulomas | Fibrosing HP |
| 17-13289 | 64 | F | Radiologic UIP, clinical possible EAA | OLB | UIP | Giant cells | Fibrosing HP |
| 17-2728 | 60 | M | Lung fibrosis | OLB | UIP | Granulomas LIP | Fibrosing HP |
| 17-46007 | 71 | M | Basal/dorsal dominant interstitial lesion, bronchiectasis | CB | UIP | LIP, granulomas | Fibrosing HP |
| 17-60122 | 47 | M | Chronic EAA | OLB | UIP | LIP granulomas CD8>4 | Fibrosing HP |
| 17-7651 | 53 | M | No response to steroids, no other data | OLB | UIP | LIP | Fibrosing HP |
| 11-106939 | 46 | F | Sarcoidosis, LAM? | OLB | fibrosis | granulomas | Fibrosing HP |
| 14-102522 | 62 | F | Mediastinal expansion | OLB | IP chron fibrosis | Granulomas, ConBr | Fibrosing HP |
| 15-41171 | 49 | F | COP, infection? | OLB | OP | LIP granulomas | Fibrosing HP |
| 15-54885 | 66 | M | ILD? probably pneumoconiosis; work in dental laboratory? | OLB | OP | LIP granulomas | Fibrosing HP |
| 15-98284 | 59 | M | Wegener’s granulomatosis? | OLB | OP | granulomas | Fibrosing HP |
| 16-42173 | 54 | F | Gas exchange disturbed, no clinical data for rheumatoid disorder or EAA | OLB | OP | LIP follic Br | Fibrosing HP |
| 16-7148 | 29 | M | Lung fibrosis? | OLB | ACIF | LIP | Fibrosing HP |
| 16-92976 | 30 | F | EAA | OLB | fibrosis | LIP granulomas | Fibrosing HP |
| 17-74534 | 29 | F | Radiologic COP | CB | UIP | LIP follic Br | Fibrosing HP |
| 14-88272 | 44 | M | Bronchiolitis obliterans, Lymphoma | OLB | UIP | Lymphocytic, giant cells | HP/ desferox? |
| 14-8664 | 62 | M | Nodules in lung, granulomatosis? | OLB | OP | LIP | HP/ drug? |
|  |  |  |  |  |  |  |  |
| 18-58284 | 79 | F | UIP? fibrosis | CB | UIP | LIP | AID? HP? |
| 17-101726 | 73 | M | Suspicious for Wegener’s granulomatosis, seronegative | OLB | UIP | Lymphocytic aspiration | AID?HP? |
| 18-33123 | 74 | F | ILD, fibrosis | OLB | UIP | lymphocytic | AID HP? |
| 12-85582 | 50 | F | Lung fibrosis, Mendelsson syndrome | OLB | UIP | Lymphocytic chron pleuritis | AID/HP |
| 17-90166 | 71 | M | ILD? | OLB | UIP | Follic br | AID/HP |
| 12-6882 | 66 | M | ANA+ ANCA-, polymyalgia | OLB | UIP | LIP | HP AID? |
| 15-65650 | 77 | F | IPF? myelodysplastic syndrome, hemochromatosis | OLB | UIP | LIP chron pleuritis | HP? AID? |
|  |  |  |  |  |  |  |  |
| 06-82508 | 64 | M | Lung fibrosis, IPF? | OLB | UIP |  | IPF |
| 07-12530 | 59 | M | Lung fibrosis, later on colon carcinoma 2011 | OLB | UIP |  | IPF |
| 07-72031 | 74 | M | lung fibrosis, IPF? | OLB | UIP |  | IPF |
| 08-137260 | 71 | F | breast carcinoma, radiation 30y ago | OLB | UIP |  | IPF |
| 08-82525 | 65 | M | ILD | CB | UIP |  | IPF |
| 12-52355 | 76 | M | UIP/IPF? | CB | UIP |  | IPF |
| 13-80491 | 61 | F | Reticulo-nodular infiltrates, granulomas, fibrosis | CB | UIP |  | IPF |
| 14-19594 | 63 | M | COP? not typical of UIP/IPF | OLB | UIP |  | IPF |
| 18-25304 | 75 | M | UIP/IPF? | OLB | UIP |  | IPF |
| 11-82507 | 69 | M | ILD? | OLB | UIP |  | IPF |
| 15-87066 | 60 | M | Clinically and radiologically not ILD | CB | UIP | lymphocytic | IPF |
| 20-84254 | 78 | M | UIP |  | UIP |  | IPF |
| 20-69594 | 68 | M | ILD, UIP |  | UIP |  | IPF |
| 20-58004 | 79 | M | ILD, IPF? |  | UIP |  | IPF |
| 20-39956 | 72 | F | UIP IPF |  | UIP |  | IPF |
| 20-32122 | 78 | M | UIP IPF |  | UIP |  | IPF |
| 19-66621 | 74 | M | UIP IPF |  | UIP |  | IPF |
| 19-64398 | 69 | M | UIP IPF |  | UIP |  | IPF |
| 19-61132 | 82 | M | UIP IPF |  | UIP |  | IPF |
| 19-34902 | 54 | M | ILD, lung fibrosis |  | UIP |  | IPF |
| 18-25304 | 75 | M | UIP IPF |  | UIP |  | IPF |
| 15-76923 | 65 | F | UIP IPF |  | UIP |  | IPF |
| 15-2194 | 61 | M | UIP IPF |  | UIP |  | IPF |
| 14-88640 | 60 | M | UIP IPF |  | UIP |  | IPF |
|  |  |  |  |  |  |  |  |
| 13-43588 | 56 | M | Honeycombing, ILD, UIP/IPF? | CB | UIP | Lymphocytic, BALT, granulomas | IPF? AID? |
| 18-72 | 73 | M | EAA/IPF | OLB | UIP | Lymphocytic chron pleuritis | AID? IPF? |
