# Supplementary material for: Lung fibrosis in autoimmune diseases and hypersensitivity: how to separate these from idiopathic pulmonary fibrosis
Source: Rheumatol Int. 2021 Oct 4;42(8):1321–30. doi: 10.1007/s00296-021-05002-2 (PMC9287245; doi:10.1007/s00296-021-05002-2)
Supplement: Supplementary file 3 — Supplementary file3 (DOCX 20 KB) [file 296_2021_5002_MOESM3_ESM.docx]

Suppl.Table 2: Definition of patterns in fibrosing pneumonia and morphologic features seen in autoimmune disease and hypersensitivity pneumonia

|  | Constant features |
| --- | --- |
| UIP | Myofibroblastic foci, cystic remodeling of primary and secondary lobules, temporal heterogeneity with uninvolved lung, spatial heterogeneity with peripheral accentuation, focal fibrosis |
| NSIP | Fibrosis of alveolar septa in fibrosing variant with remnants of lympho-histiocytic infiltrates, widening of alveolar septa, no remodeling |
| OP | Intraalveolar granulation tissue, newly formed capillaries, inflammatory infiltrate predominantly macrophages, residual leukocytes |
| ACIF | Fibrosis extending from bronchi/bronchioles into the lobules, muscular hyperplasia, metaplasia of bronchial epithelia |
| LIP | Monomorphic lympho-plasmocytic infiltrates, scattered immunoblasts, hyperplasia of BALT (bronchus associated lymphoid tissue), chronic bronchitis/bronchiolitis, follicular bronchiolitis |
| Additional features in AID and HP | Epithelioid and/or histiocytic granulomas, amyloid or immune complex deposition, ± complement activation, hemorrhage, concentric bronchiolitis with wall fibrosis |
