# Supplementary material for: Lung fibrosis in autoimmune diseases and hypersensitivity: how to separate these from idiopathic pulmonary fibrosis
Source: Rheumatol Int. 2021 Oct 4;42(8):1321–30. doi: 10.1007/s00296-021-05002-2 (PMC9287245; doi:10.1007/s00296-021-05002-2)
Supplement: Supplementary file 4 — Supplementary file4 (DOCX 22 KB) [file 296_2021_5002_MOESM4_ESM.docx]

Suppl.Table 3: Classification of cases and presence of different patterns, including combinations thereof; abbreviations: AID = autoimmune disease, HP = hypersensitivity pneumonia, IPF = idiopathic pulmonary fibrosis, , UIP = usual interstitial pneumonia, NSIP = nonspecific interstitial pneumonia, OP = organizing pneumonia, BALT = bronchus associated lymphoid tissue, ACIF = airway centered interstitial fibrosis

| Diagnosis | Number of cases |
| --- | --- |
| AID | 51 |
| fibrosing HP | 29 |
| IPF | 24 |
| Unclassified with respect to AID/HP/IPF | 9 |
| **Combinations of patterns in AID** |  |
| UIP only | 19 |
| UIP and OP | 2 |
| UIP and LIP | 9 |
| NSIP fibrosing | 1 |
| OP only | 8 |
| OP and LIP | 2 |
| unspecific fibrosis, sometimes combined with LIP | 9 |
| BALT Hyperplasia only | 1 |
| **Combinations of patterns in HP** |  |
| UIP only | 7 |
| UIP and LIP | 12 |
| OP only | 1 |
| OP and LIP, HP | 4 |
| unspecific fibrosis; in three combined with LIP | 4 |
| ACIF | 1 |
| **Specified AID** |  |
| Rheumatoid arthritis | 10 |
| Systemic sclerosis | 10 |
| Sjogren | 2 |
| Systemic Lupus | 2 |
| Dermatomyositis | 1 |
| Behcet | 1 |
| Goodpasture | 1 |
| AID probably associated with autoimmune liver disease | 1 |
| **AID versus HP** | 7 |
| **AID versus IPF** | 2 |
